# Cross-Talk Between Histone Methyltransferases and Demethylases Regulate REST Transcription During Neurogenesis

**DOI:** 10.3389/fonc.2022.855167

**Published:** 2022-05-06

**Authors:** Jyothishmathi Swaminathan, Shinji Maegawa, Shavali Shaik, Ajay Sharma, Javiera Bravo-Alegria, Lei Guo, Lin Xu, Arif Harmanci, Vidya Gopalakrishnan

**Affiliations:** ^1^ Department of Pediatrics, University of Texas, MD Anderson Cancer Center, Houston, TX, United States; ^2^ Quantitative Biomedical Research Center, Department of Population & Data Sciences, University of Texas Southwestern Medical Center, Dallas, TX, United States; ^3^ Center for Precision Health, School of Biomedical Informatics, The University of Texas Health Science Center, Houston, TX, United States; ^4^ Department of Molecular and Cellular Oncology, University of Texas, MD Anderson Cancer Center, Houston, TX, United States; ^5^ Brain Tumor Center - University of Texas, MD Anderson Cancer Center, Houston, TX, United States; ^6^ Center for Cancer Epigenetics - University of Texas, MD Anderson Cancer Center, Houston, TX, United States; ^7^ MD Anderson-UTHealth Science Center Graduate School of Biomedical Sciences, Houston, TX, United States

**Keywords:** REST (RE-1 silencing transcription factor), PRC2 (Polycomb repressive complex 2), Mll4 (KMT2D), G9a/Glp complex, KDM7A

## Abstract

The *RE1* Silencing Transcription Factor (REST) is a major regulator of neurogenesis and brain development. Medulloblastoma (MB) is a pediatric brain cancer characterized by a blockade of neuronal specification. *REST* gene expression is aberrantly elevated in a subset of MBs that are driven by constitutive activation of sonic hedgehog (SHH) signaling in cerebellar granular progenitor cells (CGNPs), the cells of origin of this subgroup of tumors. To understand its transcriptional deregulation in MBs, we first studied control of *Rest* gene expression during neuronal differentiation of normal mouse CGNPs. Higher *Rest* expression was observed in proliferating CGNPs compared to differentiating neurons. Interestingly, two *Rest* isoforms were expressed in CGNPs, of which only one showed a significant reduction in expression during neurogenesis. In proliferating CGNPs, higher MLL4 and KDM7A activities opposed by the repressive polycomb repressive complex 2 (PRC2) and the G9A/G9A-like protein (GLP) complex function allowed *Rest* homeostasis. During differentiation, reduction in MLL4 enrichment on chromatin, in conjunction with an increase in PRC2/G9A/GLP/KDM7A activities promoted a decline in *Rest* expression. These findings suggest a lineage-context specific paradoxical role for KDM7A in the regulation of *Rest* expression in CGNPs. In human SHH-MBs (SHH-α and SHH-β) where elevated REST gene expression is associated with poor prognosis, up- or downregulation of *KDM7A* caused a significant worsening in patient survival. Our studies are the first to implicate KDM7A in *REST* regulation and in MB biology.

## Introduction

The *RE1* Silencing Transcription Factor (REST), also known as Neuron Restrictive Silencing Factor (NRSF) is a major regulator of neurogenesis and brain development (1–3). It is a zinc finger transcriptional repressor and binds a consensus binding motif called the *RE1* element found in the regulatory regions of a number of neuronal differentiation and neurosecretory genes including sodium voltage-gated channel alpha subunit 2A (SCN2A), synaptophysin (SYP), synapsin-1 (SYN1), neurofilament medium polypeptide (NEFM), neurofilament light polypeptide (NEFL), microtubule associated protein-2 (MAP2), and RNA-binding protein, FOX1 homolog-3 (RBFOX3/NEUN) (4–6). More recently, we identified Ubiquitin specific peptidase-37 (*USP37*) and Patched homolog 1 (*PTCH1*), genes with roles in cell proliferation, as REST targets (7, 8). REST has also been shown to regulate the expression of, and or interact with, various non-coding RNAs including microRNAs (miRs) and long non-coding RNAs to control post-transcriptional processes including RNA processing, editing and trafficking (9–13). It is a canonical repressor, although it has also been described as a transcriptional activator in specific contexts (14). Transcriptional repression by REST requires the activity of the mSin3a and Co-REST co-repressor complexes bound to its amino (N) and carboxy (C)-termini, respectively (15, 16). Whereas mSin3A and Co-REST complexes both include histone deacetylases 1 and 2 (HDAC1/2), the Co-REST complex is characterized by the presence of the histone H3 lysine(K)-9 methyltransferase G9A and the histone H3 K-4 lysine specific demethylase, LSD1 (17–20).

REST levels are highest in pluripotent cells and in non-neural cells, where it prevents precocious or ectopic expression of neuronal differentiation genes (21). In neural progenitors, a rapid decline in REST promotes a programmed de-repression of REST target genes by mechanisms that are not well understood, to allow neurogenesis. Although, REST levels are significantly reduced in mature neurons compared to stem/progenitor cells, its maintenance at low levels is important for neuronal homeostasis and plasticity needed for repair under pathological conditions (16, 22, 23).

This homeostasis is perturbed in pediatric neural tumors such as neuroblastoma, diffuse intrinsic pontine glioma (DIPG) and medulloblastoma (24). In neuroblastoma, REST protein levels are aberrantly elevated, at least in part due to loss or decline in expression of the ubiquitin E3-ligase, SCF-β-TRCP and a failure to degrade REST protein (25). In DIPG tumors, *REST* elevation is seen in tumors with the H3K27M mutation (26). We had previously shown that *REST* expression is elevated in subgroups of patients with sonic hedgehog (SHH) medulloblastoma (MB) (1, 2). In the current study, we show that *Rest* expression is transcriptionally controlled in cerebellar granule cell progenitors (CGNPs), the cells of origin of SHH MBs.

We observed that two isoforms of *Rest* mRNA, which differed in their transcription start sites, were expressed in proliferating CGNPs and at higher levels compared to differentiated granule neurons. Onset of differentiation was associated with a significant decline in the expression of only one of the two *Rest* isoforms. In proliferating CGNPs, *Rest* mRNA expression was correlated with activity of the myeloid/lymphoid or Mixed lineage Leukemia (MLL4) and a Bromodomain and Extraterminal (BET)-domain protein at the *Rest* promoter. Repressive marks were mostly absent in the upstream regulatory region. Under differentiation conditions, the activities of MLL4 and the BET-domain protein-BRD4 were strongly countered by the combined repressive activities of the polycomb repressive complex 2 (PRC2) and the G9A/GLP complex. Pharmacological studies revealed a redundancy in the activities of these repressive chromatin remodeling complexes in silencing *Rest* expression. Our studies also uncovered a contrasting, lineage stage-specific, role for the lysine specific demethylase, KDM7A in the control of *Rest* expression in CGNPs. Consistent with this, patients with SHH-MB tumors with high REST and low or high-KDM7A expression had lower survival. 

## Results

### *Rest* Levels Are Downregulated During Neurogenesis of CGNPs

**Figure 1:**
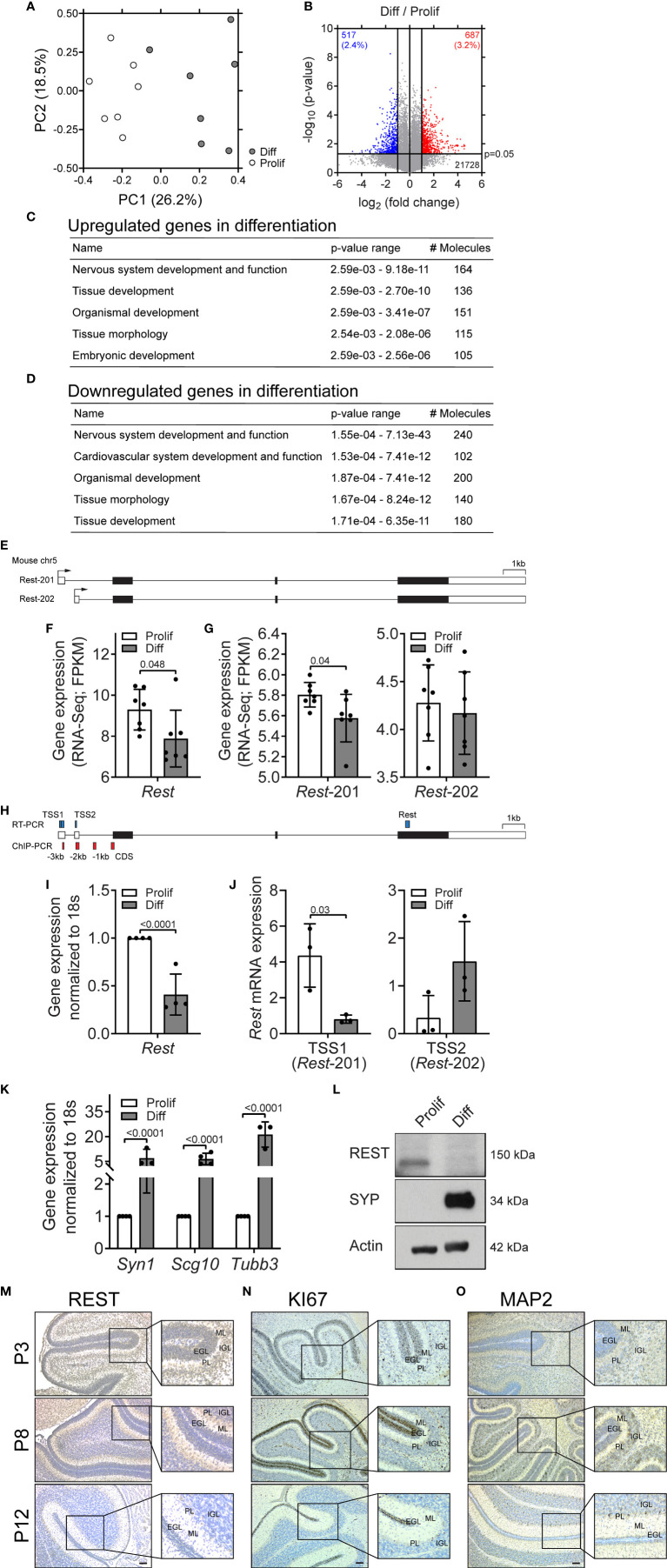
REST expression is transcriptionally downregulated during neurogenesis of CGNPs. **(A)** Principal component analysis (PCA) was performed on the RNA-Seq data derived from proliferating (white dot) and differentiating (gray dot) CGNPs. Samples are plotted using the first two principal components (PC). **(B)** Volcano plot to show fold change of gene expression between proliferating and differentiating CGNPs. The horizontal line on the y-axis indicates p-value at 0.05. **(C, D)** Top 5 enriched pathways for up-/down-regulated genes and for down-regulated genes, respectively. **(E)** *Rest* gene structure. (These includes not detectable *Rest* variants. It should be *Rest-*201 and -202.) *Rest-*201; ENSMUST00000080359.12. *Rest-*202; ENSMUST00000113449.2. **(F)** Rest expression of proliferating (white bar) and differentiating (gray bar) CGNPs by RNA-Seq (FPKM). P values were calculated based on unpaired student’s t-test. **(G)** Expression of *Rest* isoforms -201 and -202 measured by RNA-Seq (FPKM) of proliferating (white bars) and differentiating (gray bars) CGNPs. P values were calculated based on unpaired student’s t-test. **(H)** Location of PCR amplicons in Rest locus. **(I)** *Rest* mRNA levels in proliferating (white bar) and differentiating (gray bar) CGNPs. Data are means +/- SD from 5 pups for each condition. **(J)** Primers specific to TSS1 and TSS2 were used to distinguish the levels of isoforms *Rest-201* and *Rest-202* under proliferating (white bars) and differentiating (gray bars) conditions. Data shown is *Rest* expression relative to 18s. **(K)** *Syn1, Scg10* and *Tubb3* mRNA levels in proliferating (white bars) and differentiating (gray bars) CGNPs. Data are means +/- SD from 5 pups for each condition. **(L)** Western blot showing REST (proliferating) and Synaptophysin (SYP) (differentiating). Actin as a loading control is shown. Blot is representative from 2 western blots using n=5 mice for each condition. **(M–O)** Cerebellar sections from C57/Bl6 pups at P3, P8 and P12 were harvested, fixed and sectioned. Abundance of REST **(M)**, KI67 **(N)** and MAP2 **(O)** was measured by IHCs using specific antibodies. Scale bars, 20 um. EGL, external granule layer; IGL, internal granule layer; PL, Purkinje layer; ML, molecular layer.

To study regulation of *Rest* gene expression, cerebella of postnatal day 8 (P8) C57BL6 mice were harvested and cultivated in the presence of recombinant sonic hedgehog (r-SHH) for 10 days (proliferation conditions-prolif) or in the absence of r-SHH and in the presence of nerve growth factor (NGF) for 5 days (differentiation conditions-diff) (n=7, each). RNA-Seq analysis was performed followed by Principal Component Analyses (PCA) to demonstrate a clear separation of gene expression patterns in CGNPs cultured under prolif and diff conditions (**Figure 1A**). Genes that showed a significant difference in expression were subjected to pathway analysis to confirm that nervous system development and function were indeed the most altered under these conditions (**Figures 1B–D**). The expression of *Rest*, a major regulator of nervous system development, showed a significant decline (p=0.048) in gene expression during differentiation (**Figures 1E, F**). Interestingly, two isoforms (-201, -202), which differed in their transcription start site, could be mapped to the *Rest* locus under prolif conditions (**Figures 1F, G**). Of these, only *Rest*-201 showed a significant decline (p=0.04) in expression under differentiation conditions (**Figure 1G**). These data were confirmed by qRT-PCR analyses (**Figures 1H–J**) As seen in **Figure S1A**, CGNPs cultivated in the presence of r-Shh formed neurospheres, whereas their transfer to differentiation conditions caused adherence to the culture dish, development of neurite-like extensions. A decline in *Rest* levels was also confirmed by qRT-PCR analysis, which was associated with the expression of target genes such as *Syn1*, *Scg10* and *Tubb3* (**Figure 1K**). Western blotting also confirmed a decline in REST protein during differentiation (**Figure 1L**). Finally, immunohistochemistry (IHC) of cerebellar sections from P3, P8 and P12 mice (n=3), showed loss of REST staining in the external granule layer (EGL) by P12, which correlated with a decrease in KI-67 and a reduction in the thickness of this layer, and an increase in the neuronal differentiation marker, MAP2 (**Figures 1M-O**, **S1B–D**). These results indicate that *Rest* transcription, specifically that of *Rest-*201, is downregulated during neurogenesis.

### *Rest* Transcription in Proliferating CGNPs Is Maintained by Mll4

**Figure 2:**
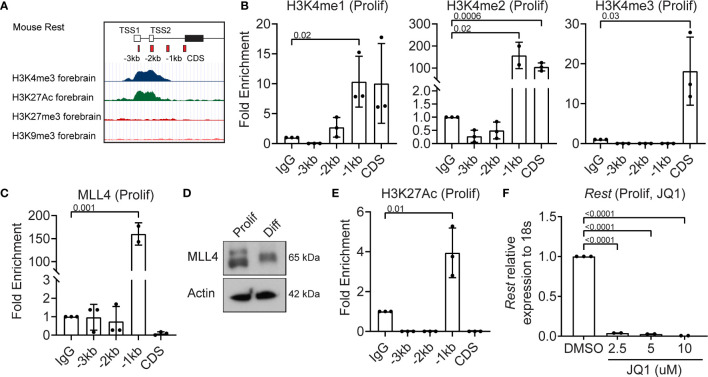
MLL4 activity is associated with transcriptional activation of REST in proliferating CGNPs. **(A)** Schematic of *Rest* locus in mouse showing H3K4me3 and H3K27me3 in the cerebellum and representing primer design strategy to include CDS and upstream promoter at -3kb, -2kb and -1kb. **(B)** H3K4me1,2 and 3 levels were measured by ChIP-qPCR and plotted as fold enrichment over IgG in proliferating progenitors. **(C)** ChIP-qPCR showing enrichment of MLL4 at the *Rest* promoter represented as fold change over IgG. **(D)** Western blot analysis of MLL4 protein and Actin from proliferating and differentiating progenitors. Shown is a representative blot from n = 2. **(E)** H3K27Ac was measured by ChIP-qPCR in proliferating progenitors and plotted as fold enrichment over IgG (n = 4 for proliferating progenitors). **(F)** Effect of BRD4 inhibition with JQ1 on *Rest* mRNA was shown by qRT-PCR in DMSO and JQ1 treated progenitors in proliferating and differentiating conditions (n = 2).

Since REST is a developmentally regulated molecule, we looked for bivalent histone modifications in the upstream regulatory region of the human REST gene in the ENCODE database. In the mouse forebrain the *Rest* URR displayed the activating H3K4me3 and H3K27me3 marks (**Figure 2A**). In the human non-neural tumor cell line, K562, a significant peak of histone H3-lysine (K)-4 trimethyl (me3) was observed at the transcriptional start site (TSS), whereas in the neural tumor cell line, NT2-D1, a peak of histone H3K27me3 was observed (**Figure S2A**). To ask if these modifications could be detected in CGNPs, chromatin immunoprecipitation (ChIP) assays were performed, using anti-histone H3K4me1, me2 and me3, and anti-MLL4 antibodies. Rabbit IgG was used as a control and for normalization. A significant level of histone H3K4me2 (p=0.0006) and me3 (p=0.025), but not me1 were detected at the start of the coding sequence (CDS) (**Figure 2B**). The -1kb region showed a significant increase in histone H3K4me1 (p=0.02) and me2 (p=0.02), but not me3 relative to IgG (**Figure 2B**). This was correlated with a 150-fold increase in MLL4 binding relative to IgG at the -1kb site (p=0.001) (**Figure 2C**). Western blotting confirmed MLL4 expression in proliferating CGNPs, although a slower migrating form of the protein was also seen under differentiation conditions (**Figure 2D**). A significant level (p=0.01) of histone H3K27 acetylation (H3K27Ac) mark was observed at the -1kb region, consistent with active *Rest* gene expression (**Figure 2E**). To ask if a JQ1-sensitive BET-domain family member (BRD2, 3, 4) was involved in reading the K3K27Ac mark, we treated proliferating CGNPs with a range of concentrations of the drug (2.5, 5, 10 μM). qRT-PCR revealed a dramatic drop in *Rest* gene expression (p<0.0001) at all 3 doses studied. These data confirmed that a JQ1-sensitive BET-domain family confirmed that BRD2, 3 or 4 was required to read the histone H3K27Ac mark (**Figure 2F**). Interestingly, RNA-Seq analyses showed that while mRNA levels for *Brd1-4* were not significantly altered between proliferation and differentiation conditions, BRD2 and BRD4 proteins were below the level of detection in proliferating CGNPs (**Figures S2B, 4D, S2C**). These data suggest that BRD2 and BRD4 proteins may be rapidly degraded, possibly to limit their activity. Similar experiments could not be performed for BRD3 due to lack of antibodies that can recognize the mouse protein.

### *Rest* Transcriptional Homeostasis in Proliferating CGNPs Is Maintained by PRC2, G9A/GLP and KDM7A

**Figure 3:**
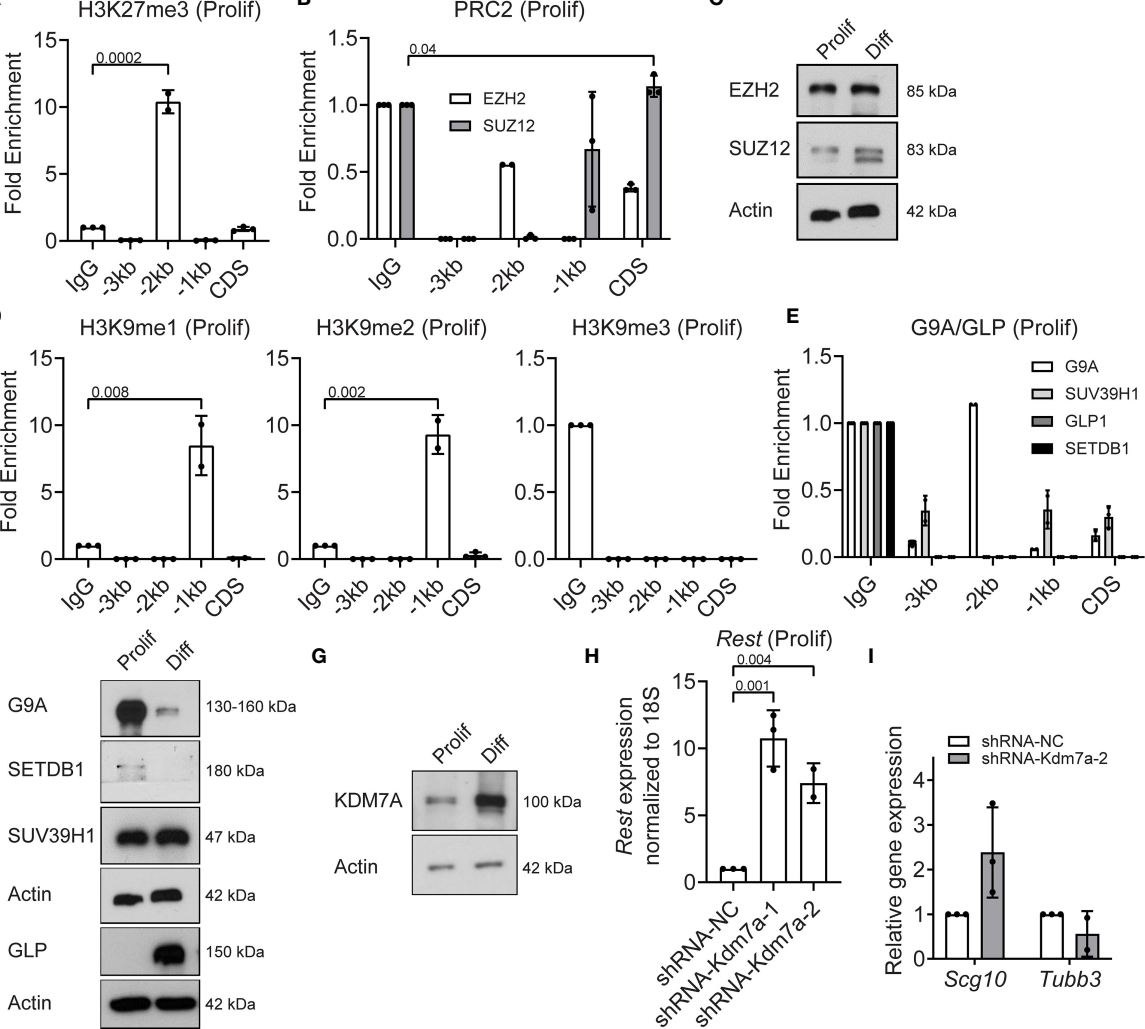
PRC2 and G9A/GLP complexes and Kdm7a repress REST expression in proliferating progenitors. **(A)** Enrichment of H3K27me3 over IgG at *Rest* promoter in proliferating CGNPs measured by ChIP-qPCR. Bars represent fold change of H3K27me3 over IgG in the samples. **(B)** Enrichment of EZH2 and SUZ12 by ChIP from proliferating progenitors were plotted as fold change over IgG in the samples. **(C)** Western blot analysis of PRC2 complex proteins EZH2 and SUZ12 from proliferating and differentiating progenitors. Data shown is a representative blot from progenitors isolated from 6 mice. **(D)** H3K9me1, 2 and 3 changes in proliferating progenitors were measured by ChIP-qPCR and represented as fold change over IgG. Bars represent fold change of H3K9me over IgG in the samples. **(E)** Enrichment of G9A, GLP, SUVAR39H1 and SETDB1 were measured by ChIP-qPCR and plotted as fold change over IgG. **(F)** Western blot showing G9A/GLP complex proteins in proliferating and differentiating progenitors normalized to Actin. Shown blot is a representative from n =2. **(G)** Western blot of KDM7A protein levels in proliferating and differentiating progenitors compared to Actin control. Shown is a representative blot from n = 2. **(H)** Knockdown of Kdm7a using shRNA specific to Kdm7a was performed and Rest transcript was measured 48 hours post shRNA addition (n=2). **(I)** mRNA levels of *Scg10* and *Tubb3* after Kdm7a knock down is shown in proliferating progenitors. For all of the ChIPs shown (unless otherwise specified) GNPs from 6 mice for proliferating conditions were pooled and the chromatin was isolated followed by IP.

Since REST is a developmentally regulated, potentially bivalent gene, we assessed levels of histone H3K27me3 deposited by the PRC2 complex. ChIP assays were performed using anti-histone H3K27me3, anti-EZH2 and anti-SUZ12 antibodies. Significant enrichment for histone H3K27me3 (p=0.0002) was noted at the -2kb region (10-fold), compared to IgG controls (**Figure 3A**). A small, but significant level of occupancy by the PRC2 complex protein, SUZ12, was observed at the CDS (**Figure 3B**). EZH2 was not bound to any of the sites examined in the *Rest* upstream regulatory region (URR) in proliferating CGNPs (**Figure 3B**). Western blot analyses confirmed the expression of EZH2 in proliferating and differentiating CGNPs, whereas SUZ12 was expressed at higher levels in differentiating cells compared to proliferating cells (**Figure 3C**). Interestingly, a distinct faster migrating SUZ12 band was seen in differentiating cells (**Figure 3C**).

Since chromatin binding of EZH2/SUZ12 was quite low and work from other groups has shown interaction between PRC2 and G9A/G9A-like protein (GLP) histone methyl transferases (27), we investigated if the latter played a role in silencing *Rest* gene expression in proliferating CGNPs. As shown in **Figure 3D**, the -1kb region showed 8-fold and 10-fold enrichment for the histone H3K9 mono (me1) (p=0.008) and dimethyl (me2) (p=0.002) marks, relative to IgG (**Figure 3D**). Histone H3K9 trimethylation (me3) was not detected at any of these sites (**Figure 3D**). Neither G9A, GLP, nor the histone H3K9 trimethyl transferases (SUV39H1 or SETDB1) showed binding at the *Rest* URR (**Figure 3E**). Western blotting revealed substantial expression of G9A and SUV39H1 and little to no expression of SETDB1 and GLP, respectively, in proliferating CGNPs (**Figure 3F**). In differentiating cells, SETDB1 was absent, G9A expression was dramatically reduced, and GLP and SUV39H1 were highly expressed (**Figure 3F**).

Histone lysine demethylases are important in the cross talk between activating and repressing complexes (28, 29). For example, the histone H3K27me3 demethylase (KDM6A), is known to be a part of the MLL4 complex, and demethylates H3K27me3 to facilitate H3K4me3 (28). However, KDM6A was not detected in proliferating CGNPs and at much higher levels under differentiating conditions (**Figure S2D**). Also, binding of KDM6A to the *Rest* URR was not seen in proliferating CGNPs (**Figure S2E**). KDM7A is a dual H3K27 and H3K9 demethylase (30, 31). KDM7A was expressed at low levels in proliferating CGNPs and higher in differentiating CGNPs (**Figure 3G**). To study its role in controlling REST expression in proliferating CGNPs, we knocked down its expression using 2 different *shRNAs* (#1 and #2). Surprisingly, KDM7A loss caused a (7.5-10-fold) increase in REST gene expression (p=0.004) in proliferating CGNPs (**Figure 3H**). Western blot revealed a 2.5-fold increase in REST protein levels under these conditions (**Figure S2F**). qRT-PCR confirmed that expression of *Scg10* and *Tubb3* expression were not significantly altered (**Figure 3I**). Collectively, the above data show that a balance between the activities of MLL4/BRD proteins and G9A-GLP/PRC2 methyltransferases/reader and the KDM7A demethylase regulate *Rest* expression in proliferation CGNPs.

### MLL4 Complex Activity Is Low at the *Rest* Locus in Differentiating CGNPs

**Figure 4:**
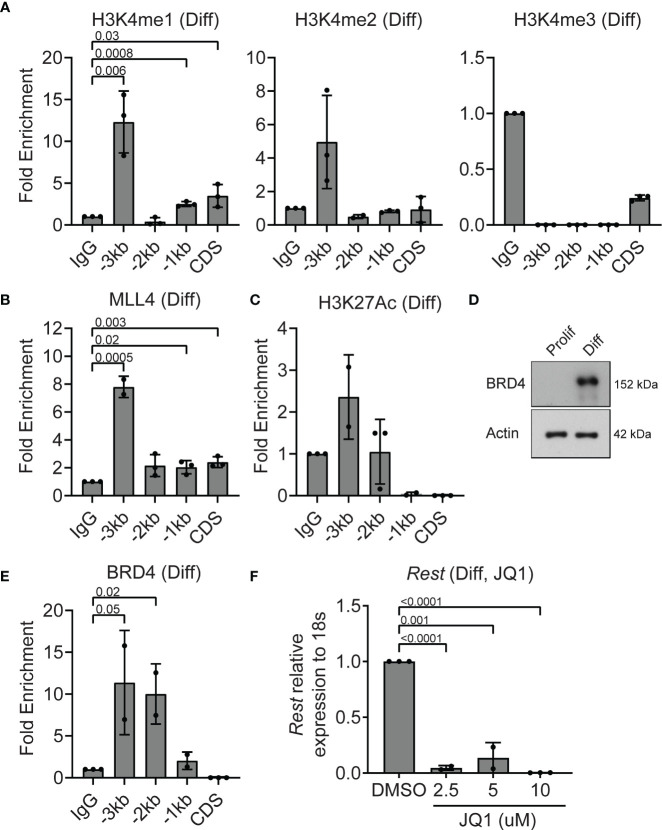
MLL4 activity at the REST locus is diminished in differentiating CGNPs. **(A)** H3K4me1, 2 and 3 levels were measured by ChIP-qPCR and plotted as fold enrichment over IgG in differentiating progenitors. **(B)** ChIP-qPCR showing enrichment of MLL4 at the REST promoter represented as fold change over IgG. **(C)** H3K27Ac was measured by ChIP-qPCR in differentiating progenitors and plotted as fold enrichment over IgG (n = 4 for differentiating progenitors). **(D)** Western blot analysis of BRD4 protein in proliferating and differentiating progenitors and ACTIN as loading control. Blot shown is a representative from n=2. **(E)** ChIP-qPCR showing enrichment of BRD4 at the REST promoter represented as fold change over IgG. **(F)** Effect of BRD4 inhibition with JQ1 on *REST* mRNA was shown by qRT-PCR in DMSO and JQ1 treated progenitors in differentiating conditions (n = 2).

To understand the epigenetic mechanisms underlying the decline in *Rest* transcription in differentiating CGNPs, ChIP assays were performed to first assess histone H3K4me3 and MLL4 occupancy at the *Rest* URR. Histone H3K4me1 was significantly enriched at -3kb (p=0.006), -1kb (p=0.0008) and CDS (p=0.03) (**Figure 4A**). Histone H3K4me2 and me3 were not detected at significant levels relative to IgG at the *Rest* URR (**Figure 4A**). A 8-fold increase in MLL4 binding to -3kb region (p=0.0005), and a small but measurable binding to the CDS (p=0.003), -1kb (p=0.02) and -2kb (p=0.06) regions was seen (**Figure 4B**). As shown in **Figure 2D**, MLL4 was expressed in differentiating CGNPs, although its migration pattern was different, suggesting the existence of one or more post-translational modification(s). Levels of H3K27Ac was elevated relative to IgG at -3kb region (**Figure 4C**). This correlated well with an increase in expression of BRD4 protein expression, and its significantly increased occupancy of the URR at -2kb (p=0.02) and -3kb (p=0.05), when compared to IgG controls (**Figures 4D, E**). The striking reduction in *Rest* expression (p<0.001) following treatment with various concentrations of JQ1 (2.5-10μM), also supports the requirement for a JQ1-sensitive BET-domain protein, potentially BRD4, in maintaining *Rest* transcription in differentiating CGNPs (**Figure 4F**).

### PRC2 and G9A/GLP Complex Activities Downregulate *Rest* Transcription in Differentiating CGNPs

**Figure 5:**
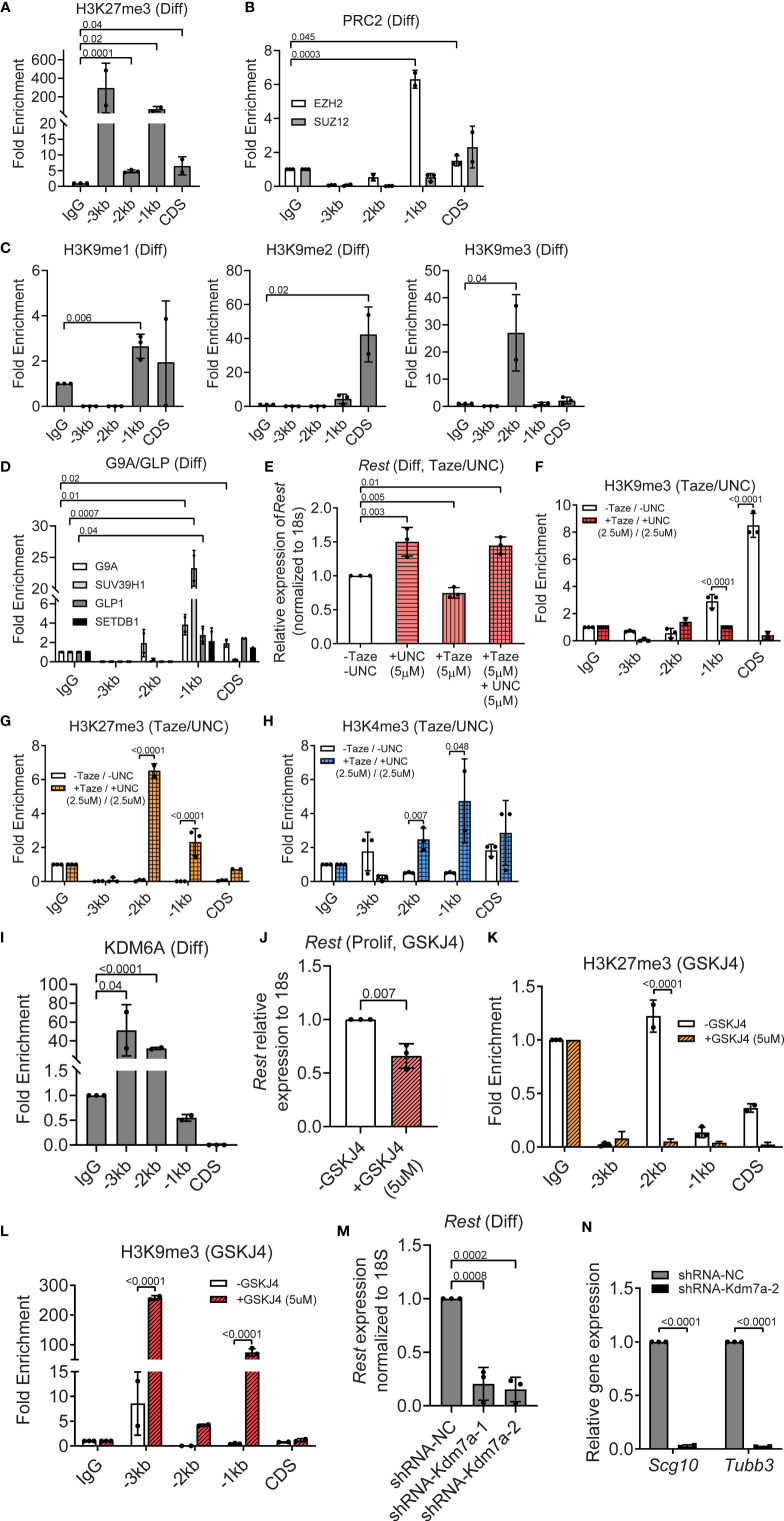
PRC2 and G9A/GLP complexes repress, whereas KDM7A activates REST expression in differentiating progenitors. **(A)** Enrichment of H3K27me3 over IgG at REST promoter in differentiating (diff) CGNPs measured by ChIP-qPCR. Bars represent fold change of H3K27me3 over IgG in the samples. **(B)** Enrichment of EZH2 and SUZ12 by ChIP from differentiating progenitors were plotted as fold change over IgG in the samples. **(C)** H3K9me1, 2 and 3 changes in differentiating progenitors were measured by ChIP-qPCR and represented as fold change over IgG. **(D)** Enrichment of G9A, GLP, and SUVAR39H1 were measured by ChIP-qPCR and plotted as fold change over IgG. **(E)** *Rest* mRNA in differentiating progenitors treated with DMSO, 5μM UNC-0638, 5μM Tazemetostat and both drugs were measured by qRT-PCR and normalized to the DMSO controls. **(F)** Change in enrichment of H3K9me3 comparing DMSO and UNC-0638 + Taze treated progenitors were measured by ChIP-qPCR and plotted normalizing the DMSO control as 1. **(G)** Differential enrichment of H3K27me3 over IgG was measured by ChIP-qPCR in DMSO and UNC-0638 + Taze treated progenitors and plotted normalizing the DMSO treated control as 1 (n = 3 for DMSO and drug treatment). **(H)** Change in enrichment of H3K4me3 comparing DMSO and UNC-0638 + Taze treated progenitors were measured by ChIP-qPCR and plotted normalizing the DMSO control as 1. **(I)** Enrichment of KDM6A at the REST promoter was measured by ChIP-qPCR and represented as fold change over IgG in proliferating and differentiating progenitors. **(J)** *Rest* mRNA was measured by qRT-PCR from DMSO and GSKJ4 treated progenitors and plotted normalizing DMSO control as 1 (n = 2). **(K, L)** Changes in H3K9me3 and H3K27me3 were measured by ChIP-qPCR in DMSO and GSKJ4 treated proliferating progenitors. Graphs were plotted by normalizing DMSO control as 1 (n = 3). **(M)** Knockdown of *Kdm7a* using shRNA specific to *Kdm7a* was performed and *Rest* transcript was measured 48 hours post shRNA addition (n = 2). **(N)** mRNA levels of *Scg10* and *Tubb3* after *Kdm7a* knock down is shown in differentiating progenitors. For all of the ChIPs shown (unless otherwise specified) GNPs from 6 mice for differentiating conditions were pooled and the chromatin was isolated followed by IP.

In contrast to histone H3K4me3, histone H3K27me3 was significantly enriched relative to IgG at the CDS (6-fold, p=0.04), -1kb (100-fold, p=0.02), -2kb (5-fold, p=0.0001) and -3kb (300-fold, p=0.13) regions of the *Rest* URR (**Figure 5A**). Expression of EZH2 and SUZ12 was readily detected in differentiating CGNPs by Western blotting (**Figure 3C**). However, only EZH2 occupancy was observed in differentiating cells, and a 2-fold (p=0.045) and 7-fold (p=0.0003) enrichment, respectively, was seen at CDS and -1kb region of the *Rest* URR (**Figure 5B**). In contrast to histone H3K27me3, enrichment of histone H3K9 methylation was strongest at the CDS and -2kb regions, whereas these marks were present at lower levels at -1kb. Specific elevation of histone H3K9me2 (40-fold, p=0.02) at the CDS, histone H39me1 at -1kb (3-fold, p=0.006) and histone H3K9me3 (30-fold, p=0.04) at -2kb of the *Rest* URR, were noted in differentiating CGNPs (**Figure 5C**). Relative to control IgG, the histone H3K9 mono- and dimethyl-transferase, G9A showed significant occupancy at the CDS and -1kb (p=0.02 and 0.01, respectively). Its heterodimeric partner, GLP and the histone H3K9 trimethyl transferase SUV39H1 showed a 3-fold (p=0.04) and an approximately 25-fold (p=0.0007) enrichment at the -1kb region (**Figure 5D**). Significant binding of SETDB1 was not found at the *Rest* URR (**Figure 5D**).

To determine the relevance of these repressive histone modifications in silencing REST transcription, differentiating CGNPs were treated for 8 hours with either 5μM Tazemetostat (Taze) – an Ezh2 inhibitor, or 5μM UNC-0638 – a G9a inhibitor, alone, or in combination (5μM each) to account for redundant use of the two repressive complexes. As shown in **Figure S3A**, treatment with Taze alone resulted in a significant decrease in H3K27me3 at -3kb (p<0.0001) and CDS (p<0.0001). At the same time, an increase in histone H3K9me3 deposition at -3kb (p=0.02) and at -1kb (p=0.002), whereas a significant reduction in this modification was noted at -2kb (p<0.0001) and CDS (p<0.0001) (**Figure S3A**). UNC-0638 treatment resulted in a significant decrease in histone H3K9me3 at -2kb (p<0.0001) and CDS (p=0.002) regions of the *Rest* URR (**Figure S3B**). This mark was significantly increased at -3kb (p=0.03) and -1kb (p<0001) (**Figure S3B**). Histone H3K27me3 was significantly reduced at -3kb (p<0.0001), -1kb (p=0.002) and CDS (p<0.0001), but elevated at -2kb (p=0.007), following UNC-0638 treatment (**Figure S3B**). These data indicated a potential for redundancy in these two repressive marks.

To test this possibility, CGNPs cultivated under differentiation conditions and co-exposed to Taze and UNC-0638, as described above. As seen in **Figure 5E**, UNC-0638 and the combination resulted in a 1.5-fold increase in *Rest* expression (p=0.003 and 0.01, respectively). Surprisingly, treatment with Taze alone promoted a significant reduction (p=0.005) in *Rest* gene expression compared to untreated controls (**Figure 5E**). The unexpectedly weak de-repression of *Rest* gene expression raised the probability of a redundant recruitment of the repressive histone H3K9me apparatus or that of EZH1 upon loss of EZH2 activity, or alternatively the requirement for an activating mechanism following loss of these repressive mechanisms, to upregulate *Rest* gene expression. Additionally, the roughly equal increase in *Rest* gene expression in the presence of UNC-0638 alone and the combination of UNC-0638 and Taze, suggested that the activity of the G9A/GLP/SUV39H1 complex may be more important for the silencing of *Rest* transcription in differentiating CGNPs. To further understand these results, ChIP experiments were performed to measure changes in histone H3K9me3 and histone H3K27me3 following treatment with both drugs (2.5μM, each). Histone H3K9me3 showed a significant reduction in enrichment at CDS (p<0.0001) and -1kb (p=0.0001) as would be expected following UNC-0638 treatment (**Figure 5F**). However, histone H3K27me3 was significantly enriched at -1kb (3-fold) and -2kb (7-fold) (p<0.0001, each) in the presence of both drugs compared to the no treatment control (**Figure 5G**). These findings suggest that an alternative mechanism, such as EZH1 recruitment may be the source of the persistent H3K27me3 at the *Rest* URR following EZH2 inhibition. A 5-fold increase in histone H3K4me3 observed at -1kb following treatment with both drugs, also suggests that the failure to promote a stronger recruitment of the MLL4 transcriptional activating machinery may contribute to the absence of a more robust upregulation of *Rest* expression (**Figure 5H**).

The demethylase KDM6A, which showed strong expression in differentiating CGNPs, exhibited significant binding at -2kb (5-fold, p<0.001) and -3kb (50-fold, p=0.04) regions of the *Rest* URR (**Figures 5I**, **S2D**). Its pharmacological inhibition by the drug GSKJ4 caused a significant downregulation of *Rest* expression (p=0.007) in differentiating CGNPs (**Figure 5J**). Surprisingly, a decrease in histone H3K27me3 (p<0.0001) at the -2kb site (**Figure 5K**), and an increase in histone H3K9me3 at -1kb (100-fold, p<0.0001) and -3kb (250-fold, p<0.0001) sites, highlight a novel cross-talk between the histone H3K9-me apparatus and the H3K27me demethylase, KDM6A (**Figure 5L**). Additionally, knockdown of KDM7A, in differentiating CGNPs caused a 5-fold downregulation of *Rest* mRNA levels 5-fold (p=0.0008, p=0.0002), and surprisingly, a significant reduction in the expression of REST target genes, *Scg10* (p<0.001) and *Tubb3* (p<0.001). These findings suggest that KDM7A, which is known to be required for neurogenesis, may control other targets required for the activation of expression of REST target genes (**Figures 5M, N**). Thus, our data with primary mouse CGNPs indicate that *Rest* mRNA homeostasis is orchestrated by the MLL4/BRD4, PRC2/G9A/GLP/SUV39H1 complexes and the demethylases-KDM6A and KDM7A. Most importantly, KDM7A had a differentiation stage-specific role in the regulation of *Rest* expression.

**Figure 6:**
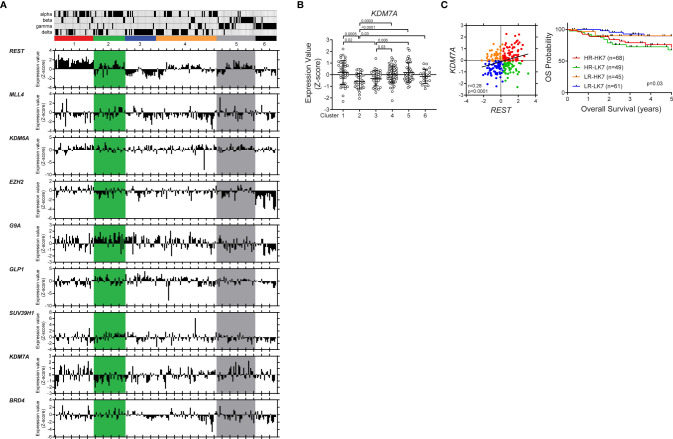
*KDM7A* up- or down-regulation is associated with significantly poor survival in patient with *REST*-elevated SHH MBs. Hierarchical clustering analysis identified six distinct clusters based on expression profiles of neuronal differentiation markers in SHH MB patient samples (www.ncbi.nlm.nih.gov/geo; dataset GSE85217, Sci Signal. 2019). Vertical column corresponds to one individual with subtype (alpha, beta, gamma, and delta). Six-colored boxes represent the six-clusters. The bar graphs show the gene expression values (Z score) of each patient. **(A)** Differential expression of *REST*, *MLL4*, *KDM6A*, *EZH2*, *G9A*, *GLP1*, *SUV39H1*, *KDM7A* and *BRD4* in the different clusters are shown. **(B)** *KDM7A* mRNA expression profile in SHH MB patient samples. Hierarchical clustering based on expression of neuronal differentiation markers divided the SHH MB patient samples into six distinct clusters (Cluster 1; n = 39, Cluster 2; n = 31, Cluster 3; n = 32, Cluster 4; n = 61, Cluster 5; n = 39, Cluster 6; n = 21). Each dot corresponds to an individual patient. Data show individual variability and means ± SD. P-values were obtained using the unpaired t-test with Welch’s correction. **(C)** Four patient groups based on *REST* and *KDM7A* expression levels. Overall survival of four patient groups based on *REST* and *KDM7A* in patients with SHH MB (P value; log-rank Mantel-Cox test).

To ask if this regulatory mechanism is perturbed in human SHH medulloblastoma tumors, a publicly available database was probed for expression of the above complex members. In our previous work, we showed the sub grouping of human SHH MBs into 6 clusters based on their neuronal differentiation status (32, 33). The distribution of the samples belonging to the published subtypes (α, β, γ, δ) between these clusters is also indicated in **Figure 6A**. It is known that patients with SHH-α and -β tumors have the worst survival (1, 3). REST expression was elevated in clusters 1, 2 and 5 (1). Here we studied the expression of the chromatin modifiers (*MLL4*, *BRD4*, *EZH2*, *SUZ12*, *G9A*, *GLP1*, *SUV39H1*, *KDM6A* and *KDM7A*) implicated in the regulation of the mouse *Rest* gene. Expression data shown in **Figure 6A** is relative to the average expression (Z-score) of each gene across all other groups of medulloblastoma samples (SHH group Clusters from (32) In cluster 2, where *REST* expression is elevated, and the tumors are more immature, a number of patients tumors consistently expressed *G9A* and *KDM7A* at levels higher or lower than the Z-score (**Figure 6A**). In cluster 5, where *REST* is elevated and the tumors are known to be more committed to neuronal differentiation, expression of the repressive enzymes- *EZH2*, *SUZ12*, *G9*A *GLP*, *SUV39H1* was lower than the Z-score (**Figure 6A**). Expression of the demethylases *KDM6A* and *KDM7A* was higher than the Z-score (**Figure 6A**). A positive correlation between *REST* and *MLL4* (p=0.03, r=0.40), *REST* and *SUV39H1* (p=0.02, r=0.43) and *REST* and *KDM7A* (p=0.001, r=0.56) was observed in cluster 2 (**Figure S4A**). In cluster 5, *REST* and *SUV39H1* were negatively correlated (p=0.046, r=-0.32), while *REST* and *KDM7A* exhibited a positive correlation (p=0.002, r=0.48) (**Figure S4A**).

**Figure 7:**
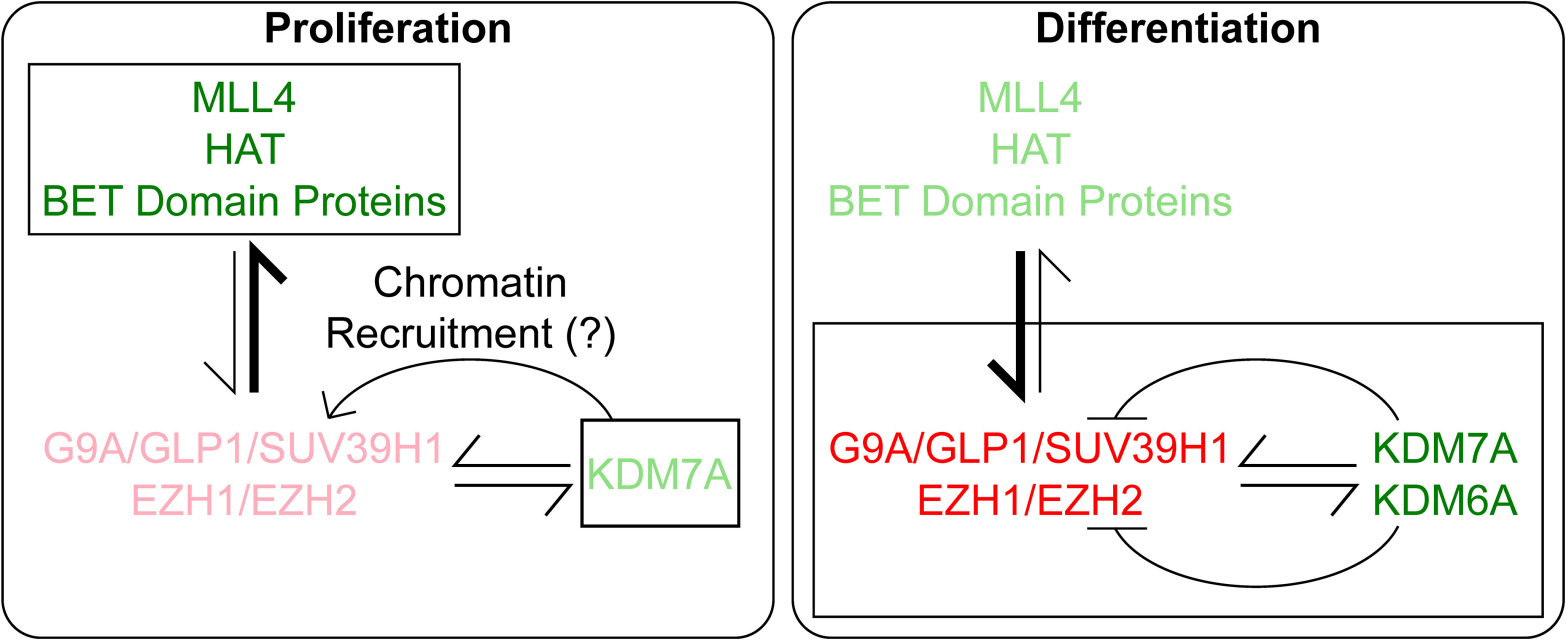
Under Proliferation conditions, *Rest* transcription is activated by MLL4, HAT and BRD proteins and negatively regulated minimally by PRC2 and demethylase, KDM7A, where we hypothesize it helps in chromatin recruitment of the repressive complexes. In contrast, during differentiation, the repressive complexes G9A and PRC2 play a major role in controlling *Rest* transcription. At the same time, a lower level of *Rest* expression is maintained by activating marks and the demethylases KDM6A and KDM7A. The level of action of the proteins are denoted by the boldness of the font, brighter denotes stronger activity and lighter font denotes lower activity.

Consistent with the above results, *KDM7A* gene expression was significantly down- and upregulated in clusters 1 and 5, respectively (**Figure 6B**). Finally, we divided SHH MBs into four groups based on the expression levels of *REST* and *KDM7A*, and found that low-*REST*/low-*KDM7A* expressions were associated with a significantly better overall survival (OS) (p=0.03) in SHH MB patients. In contrast, patients with high *REST* and low *KDM7A* or high *REST* and high *KDM7A* expression in their tumors, had the worst OS at 5 years (p=0.03), in line with seemingly opposite role of *KDM7A* on *REST* expression based on the differentiation status of the cells (**Figures 6C**, **S4B**). Thus, our results indicate a cross talk between activating and repressive modifiers during neuronal differentiation of CGNPs (**Figure 7**). These data have implications for SHH MB tumors.

## Materials and Methods

### CGNP Isolation, Proliferation and Differentiation

Cerebellar tissue from P8 pups of C57BL6 mice were dissected and triturated using 18.5 gauge needle with 10 ml syringe to form single cell suspension. CGNPs were expanded for 10 days accompanied by trituration every 4 days, in Neurobasal medium supplemented with B27, Glutamax, Antibiotic/Antimycotic, Heparin and 20ug/ml of Epidermal growth factor (EGF), Fibroblast growth factor (FGF), and then for 4 days in the presence of 0.1μg/ml recombinant mouse sonic hedgehog (rShh) (proliferation conditions) (34). Differentiation of CGNPS was achieved by culturing them in the absence of EGF and FGF and with the addition of 20μg/ml of neural growth factor (NGF) after their initial cultivation in the presence of Shh ligand as above. Differentiated cells were collected with TrypLE for further molecular analyses.

### RNA and cDNA Preparation

The pellets from proliferating and differentiated neural progenitors were used to isolate RNA using RNA mini kit in accordance with manufacturer’s instructions (Zymo Research, Irvine, CA). RNA was quantified using a Nano Drop 2000 (Thermo Fisher Scientific, MA). Equal concentrations of RNA were used to synthesize cDNA using iscript cDNA kit (Bio-Rad, Hercules, CA) and then measure gene expression.

### Immunohistochemistry

Mouse brain tissues were fixed in 10% buffered formalin phosphate and embedded in paraffin. Eight-micrometer-thick brain sections were used for IHC analysis. Sections were deparaffinized with xylene, followed by rehydration with ethanol and water. Antigen retrieval was performed in citrate antigen retrieval buffer (pH 6) for 30 minutes at 95°C in the PT module (ThermoFisher). Sections were washed with 0.1% Triton X in PBS (PBST) and then treated with 3% H2O2 solution for 10 min to block the endogenous peroxidase. Nonspecific binding of antibodies was blocked with 3% BSA in PBS for 1 hour. The sections were then incubated with primary antibodies as indicated in **Table 1**, diluted in blocking buffer, at 4°C overnight. The primary antibody was detected using a secondary antibody conjugated to horseradish peroxidase (HRP; the Jackson Laboratory) by incubating sections for 1 hour at room temperature. All incubations were performed under humidified conditions. Last, slides were washed with TBST and developed using the DAB kit (Vector laboratories) and counterstained with hematoxylin. After dehydration and mounting, slides were visualized under a Nikon ECLIPSE E200 microscope, and images were captured under 4x, 10x, 40x and 100x magnification with an Olympus SC100 camera. Analyses were performed using Olympus cellSens Entry software.

**Table 1:** Antibodies for IHC.

Antibody	Cat. #	Company	Dilution
REST	HPA006079	Sigma-Aldrich	1:75
Ki-67	ab15580	Abcam	1:100
MAP2	AB5622	Millipore Sigma	1:100
anti-rabbit-HRP	111-035-003	Jackson ImmunoResearch	1:200

### RNA-Seq and PCA Analyses

RNA was harvested from pooled WT CGNPs and quality checked using RNA Nano assay using Bioanalyzer 2100 (Agilent Technologies - RNA RIN should be >8). Quantification was done using Qubit 2.0 Fluorometer (Thermo Fisher Scientific, Waltham, MA). Library preparation was done with 1000 ng of RNA using TruSeq Stranded Total RNA Gold kit (Illumina RS-122-2303) following TruSeq Stranded Total RNA Reference guidelines. RNA-Seq library quality/quantity was checked using Tape Station 2200 D1000 assay (Agilent Technologies, Santa Clara, CA and QuantStudio 6 Flex Real time PCR System (Applied Biosystem, Waltham, MA), and sequenced (paired end-75 cycles) using Hiseq 3000 (Illumina, San Diego, CA). The raw sequencing reads were aligned to the human GRCm39 genome (Star Aligner version 2.7.3a) and quantified using featureCounts (subread v1.6.3) based on Ensembl gene model GRCm39 vM27. Genes with count per million (CPM) greater than 0 in all sample were selected and normalized using the voom method of the R package limma v3.50.0 (35). Batch effects were removed using the R package sva v3.42.0 (36). Differential gene expression analysis was performed using the R package limma v3.50.0. Principal component analysis was performed for the samples in R v4.1.1.

### Pathway Analyses

Functional class annotation analysis was performed on up/down-regulated genes by using the Ingenuity Pathway Analysis software. We analyzed biological processes, molecular functions, and cellular components that were relatively enriched by the gene lists of interest.

### Quantitative Reverse Transcriptase PCR

Quantitative RT-PCR was performed in triplicate using 2x SYBR-Green master mix (Bioline, Boston, MA) and run on a LightCycler 96 Real-Time PCR System (Roche Diagnostics GmbH, Mannheim, Germany). Relative mRNA expression was calculated using mouse 18S RNA as a control. The sequence of the primers used are listed in **Table 2**.

**Table 2:** Primers for qRT-PCR.

*Rest*	5’-CGCCGTCAGCAACGAGAAGAT-3’	5’-GCTTTAGTCTCCGCCGCCAC-3’
*Tubb3*	5’-CCCTTCGATTCCCTGGTC-3’	5’-ACGGCACCATGTTCACAG-3’
*Syn1*	5’-GGACGGAAGGGATCACATTATT-3’	5’-ACCACAAGTTCCACGATGAG-3’
*Scg10*	5’-GCGCAACATCAACATCTACAC-3’	5’-AGATGGTGGCTTCAAGATCAG-3’
*18s*	5’-GCAATTATTCCCCATGAACG-3’	5’-GGCCTCACTAAACCATCCAA-3’

### Protein

Protein lysates from CGNPs were prepared in EBC lysis buffer (50 mM Tris, pH 8.0, 120 mM NaCl, and 0.5% NP-40) in the presence of protease inhibitors (Thermo Fisher Scientific, Waltham, MA) and phosphatase inhibitor cocktail-2 (Sigma, St. Louis, MO). Cleared lysates were used to measure protein concentration in supernatants using Bio-Rad protein assay dye reagent (Bio-Rad Laboratories, Hercules, CA). Samples were run on a SDS-PAGE and Western blot analyses were performed using primary antibodies against the proteins indicated in the table (**Table 3**). Membranes were washed and incubated with the corresponding HRP-conjugated secondary antibodies (Jackson Immuno Research, West Grove, PA), and developed using SuperSignal (Cat#34075; Cat#34087; Thermo-Scientific, Waltham, MA) followed by autoradiography.

**Table 3:** Antibodies for Western Blot.

Antibody	Cat. #	Company	Dilution
REST	07-579	EMD Millipore	1:800
MLL4	ab104444	Abcam	1:1000
EZH2	5246S	Cell Signaling	1:1000
SUZ12	3737S	Cell Signaling	1:1000
G9A	ab185050	Abcam	1:1000
GLP	PP-B0422-00	R&D Systems	1:1000
UTX	33510	Cell Signaling	1:1000
ACTIN	4967S	Cell Signaling	1:10000
BRD4	A301-985A-M	Bethyl Laboratories	1:1000

### Chromatin Immunoprecipitation Assay

GNPs from proliferating and differentiating cells were fixed with formaldehyde, cross-linked, and processed for chromatin immunoprecipitation (ChIP) analyses as described previously (15). Cross-linked cells were resuspended in sonication buffer (50 mmol/L Tris-HCl (pH 8.0), 10 mmol/L EDTA (pH 8.0), 1% SDS, and protease inhibitors) and sonicated, and 10% of this material was saved as input DNA. The remainder of the samples was diluted 5-fold with ChIP dilution buffer [16.7 mmol/L Tris-HCl (pH 8.0), 167 mmol/L NaCl, 1.2 mmol/L EDTA (pH 8.0), 1.1% Triton X-100, and protease inhibitors], precleared, and incubated with various antibodies (**Table 4**) overnight at 4°C. Following incubation with protein-A beads, washing, and elution, the cross-linking was reversed, and DNA was purified with a PCR Purification Kit (Zymo). Bound DNA was quantified by SYBRGreen qPCR using the listed primers using a Roche Lightcycler 96. Data were analyzed using the comparative 2−ΔΔCt method. Primers are listed in **Table 5**.

**Table 4:** Antibodies for ChIP.

Antibody	Cat. #	Company
REST	07-579	EMD Millipore
Tri-Methyl H3K27	ab6002	Abcam
Tri-Methyl H3K4	4658	Cell Signaling Technology
Tri-Methyl H3K9	13969S	Cell Signaling Technology
EZH2	5246S	Cell Signaling Technology
SUZ12	3737S	Cell Signaling Technology
MLL4	ab104444	Abcam
G9A	ab185050	Abcam
GLP	ab41969	Abcam
SUVAR39H1	ab155164	Abcam
IgG	sc-2027	Santa Cruz Biotechnology
Acetyl H3K27	8137s	Cell Signaling Technology
BRD4	A301-985A100	Bethyl Laboratories

**Table 5:** Primers for ChIP.

*CDS*	5’-TTGCATTGGAAAGATGCTTGCT-3’	5’-AAGAGACTGCCTCCTCCAGA-3’
*-1 kb*	5’-TTGCAAGAGGCACACTGAGTT-3’	5’-AACTTAGCCGCATTGTGCTTG 3’
*-2 kb*	5’-GTTCGCAAACTTCCGGGC-3’	5’-AAAGTTACCTCGCTACCCGC-3’
*-3 kb*	5’-GCCTCGACGCCCAACTT-3’	5’-CTTTTCTTTGTTGCTGGACTTTC-3’

### *Kdm7a* Knockdown

3-5 million proliferating and differentiating progenitors were used for shRNA assay. Both proliferating and differentiating progenitors were treated with either nonsense control shRNA (shRNA-NC) or two different shRNA constructs targeting *Kdm7a*. 500μl of the virus was added to 1.5ml of cells in media and incubated overnight at 34°C. The cells were then moved to 37°C incubator and the media was changed after 24 hours and allowed to grow. After 72 hours of shRNA introduction, cells were harvested and RNA extraction was performed using Quick RNA mini kit (Zymo research). cDNA was synthesized using Iscript reverse transcriptase kit (Biorad, USA). RT-PCR was performed to analyze knockdown of *Kdm7a*, *REST* and normalized using *18S* as a control. Data were analyzed using the comparative 2−ΔΔCt method.

## Discussion

Regulation of REST protein through proteasome pathway has been fairly well defined through work from a number of groups, including ours (16, 32, 37). Since our previous work described the deregulation of *REST* mRNA levels in SHH-driven MBs, we sought to understand the control of its gene expression using murine CGNPs as a model system. Our studies described here identified the expression of two isoforms of *Rest* in mouse CGNPs, that differed in their transcription start site. Interestingly, only one of the two isoforms (*Rest*-201) was significantly downregulated during neurogenesis, with *Rest*-202 likely facilitating the maintenance of *Rest* transcript at low levels essential for neuronal survival. Given that these isoforms differ only in their 5’untranslated region (5’UTR), CAP-dependent or independent translation may be key to protein synthesis from these transcripts, a possibility that remains to be addressed (38). The differential expression of these isoforms in human SHH MBs and their contribution to tumorigenesis are also of future research interest.

Our studies also provide the first description of the epigenetic mechanisms underlying the downregulation of *Rest* mRNA during neurogenesis of CGNPs. Developmentally controlled genes are frequently bivalent genes in that their promoters are characterized by a basal level of both trimethylation at H3K27 and at H3K4 residues. Depending on the signals received to either turn on or off gene expression, levels of K27 trimethylation and K4 trimethylation are modulated. Consistent with *REST* being a developmentally regulated gene, histone H3K4me3 and histone H3K27me3 marks were seen at the *Rest* URR. Interestingly, the presence of the histone H3K9 methylation suggests the existence of H3K4me3-H3K9me2/3 bivalency at this locus and that coordinated activities of both repressive marks may allow for a tight regulation of *Rest* expression in proliferating CGNPs (39–41).

We have also defined a complex interplay between activating and repressive chromatin remodeling activities to allow *Rest* transcriptional homeostasis in neural progenitors and in differentiating neurons. While the need for MLL4 appears to be established for the activation of *Rest* expression, we uncovered a neuronal differentiation-stage specific requirement for the JQ1-sensitive BET-domain family of proteins. The sensitivity of proliferating and differentiating CGNPs to JQ1, despite the absence of detectable BRD2 and BRD4 proteins raises the possibility that these proteins, if relevant to the control of Rest transcription in proliferating CGNPs, may be rapidly turned over. A role for BRD3 can be ruled out, but the lack of an antibody than can recognize mouse BRD3 disallowed further investigations (42, 43). While the activities of the above complexes predominate in proliferating CGNPs, a lower level of repressive histone modifications deposited by the PRC2 and G9A complexes serve to keep MLL4 activity in check at the *Rest* URR.

Interestingly, the process of neurogenesis is associated with a switch in the role of these complexes, with the repressive complexes taking on a more dominant role and potentially limiting MLL4 enrichment at the chromatin. In addition, an alternative isoform or its post-translational modification, possibly accounting for altered protein migration in differentiating CGNPs, could explain the lack of DNA binding activity in differentiating cells, which remains to be investigated in future studies. The observation that Taze, a drug with strong activity against EZH2, failed to ablate H3K27me3 in the *Rest* URR, suggests that EZH1 may play a role in compensating for loss of EZH2 activity. Indeed, a recent report identified a proteomic network involving EZH1, EZH2, G9A, GLP and SUV39H1 (44).

Of considerable interest to us was our discovery of a role for the histone H3K27me demethylase-KDM6A, and the histone H3K27me/K9me demethylase-KDM7A in the regulation of *Rest* transcription. In fact, the requirement for KDM7A was strikingly divergent in proliferating and differentiating CGNPs. Whereas its role as a demethylase may explain the downregulation of *Rest* expression following its knockdown in differentiating cells, the upregulation of *Rest* mRNA levels following its loss in proliferating CGNPs suggests a more indirect role, such as recruitment of the PRC2 complex to chromatin (45). These findings have clinical relevance because loss or increase in *KDM7A* expression can drive *REST* elevation, which we have previously shown to be a driver of MB genesis in genetically engineered mice and xenograft models (8, 32, 46). Here, understanding the mechanism that controls the transcription of *REST* during normal neurogenesis and comparing it with disease states has identified novel opportunities for therapeutic targeting (32). For example, the current study suggests that KDM7A may be a novel therapeutic target. However, the duality of KDM7A function in REST regulation, which is reflected in the poor survival associated with its up-or down-regulation in REST-elevated SHH MBs, advocates for a more calibrated approach to the design of pre-clinical studies. BET-domain inhibitors may hold more promise to pharmacologically decrease REST levels in tumors.

## Data Availability Statement

The datasets presented in this study can be found in online repositories. The names of the repository/repositories and accession number(s) can be found below: National Center for Biotechnology Information (NCBI) BioProject database under accession number GSE196548.

## Ethics Statement

The animal study was reviewed and approved by The University of Texas MD Anderson Cancer Center’s Animal Care and Use Committee.

## Author Contributions

JS, SM: Conception, experimental design and execution of work, writing and manuscript edits; SS, JB-A: experimental design and execution of work and manuscript edits; AS: reagent generation; LG: bioinformatic analysis and execution of work, writing and manuscript edits; LX: bioinformatics, oversight of work, manuscript edits, funding support; AH: bioinformatics; VG: Conception, experimental design, oversight of work, writing and manuscript edits, funding support. All authors contributed to the article and approved the submitted version.

## Funding

This work was supported by grants to VG from the Cancer Prevention Research Institutes of Texas (CPRIT-RP150301), NIH (R01NS079715 and R03NS077021) and Addi’s Faith Foundation.

## Conflict of Interest

The authors declare that the research was conducted in the absence of any commercial or financial relationships that could be construed as a potential conflict of interest.

## Publisher’s Note

All claims expressed in this article are solely those of the authors and do not necessarily represent those of their affiliated organizations, or those of the publisher, the editors and the reviewers. Any product that may be evaluated in this article, or claim that may be made by its manufacturer, is not guaranteed or endorsed by the publisher.
