# Supplementary material for: Cross-Talk Between Histone Methyltransferases and Demethylases Regulate REST Transcription During Neurogenesis
Source: Front Oncol. 2022 May 6;12:855167. doi: 10.3389/fonc.2022.855167 (PMC9120943; doi:10.3389/fonc.2022.855167)
Supplement: Supplementary file 1 [file DataSheet_1.docx]

**
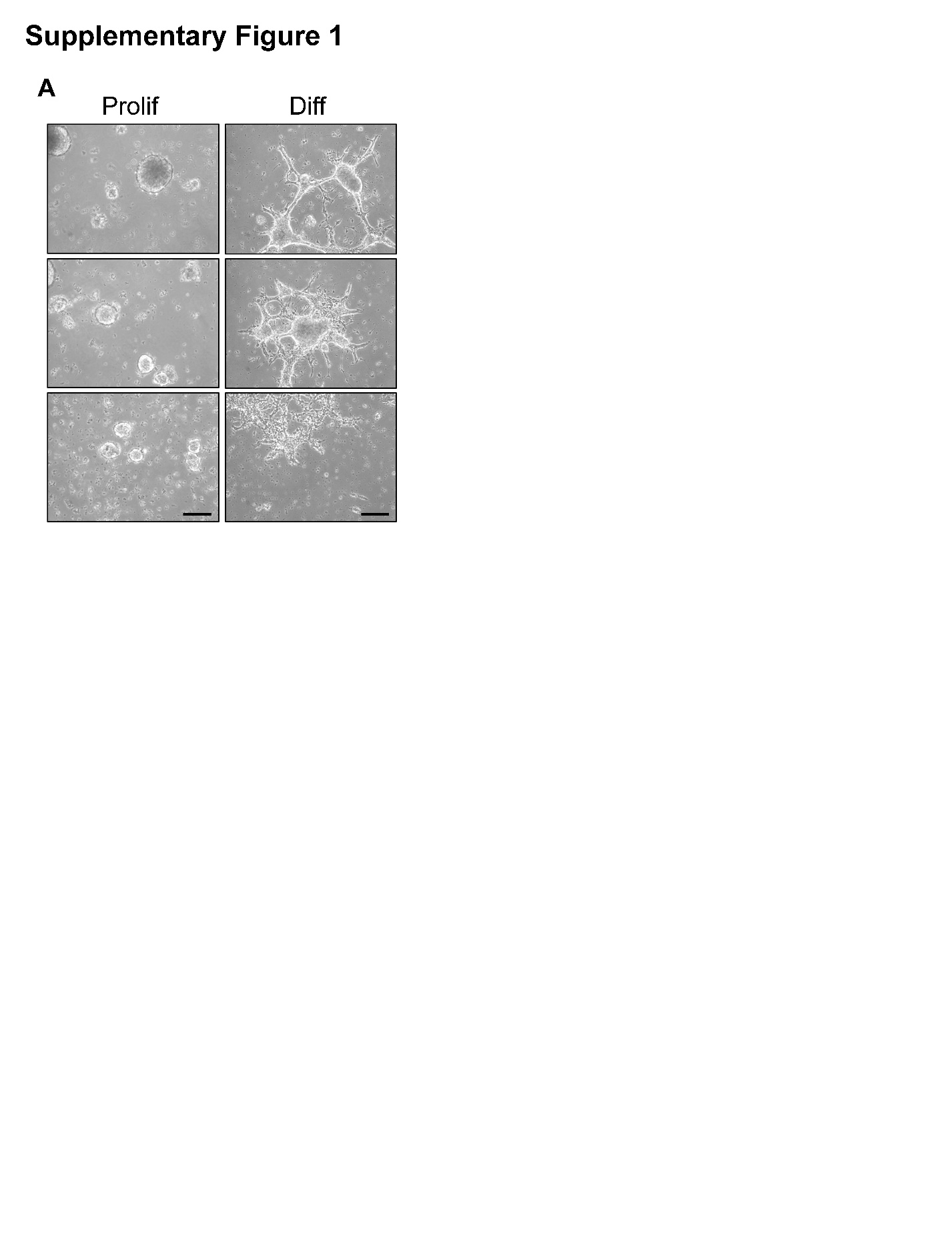
**

**
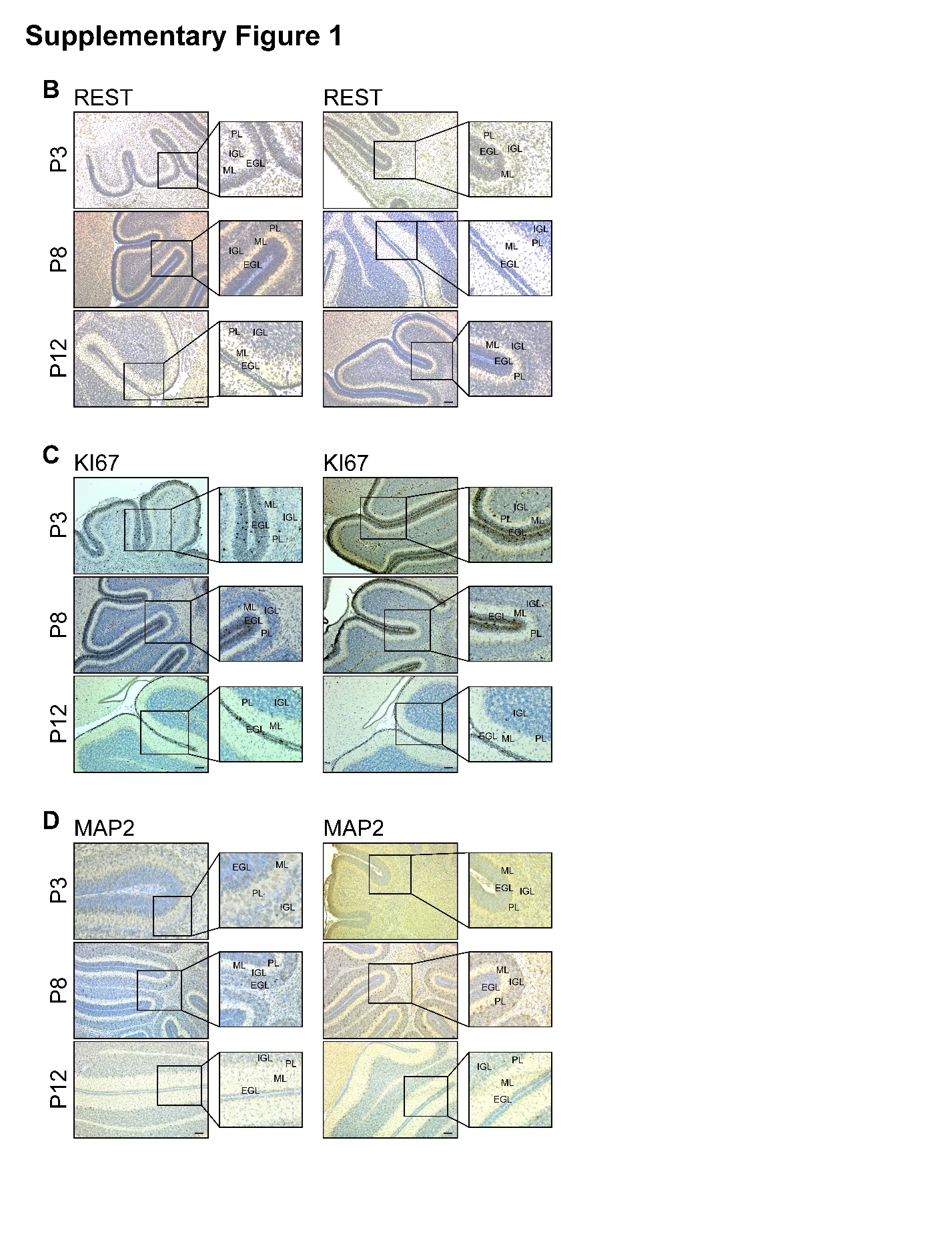
**

**
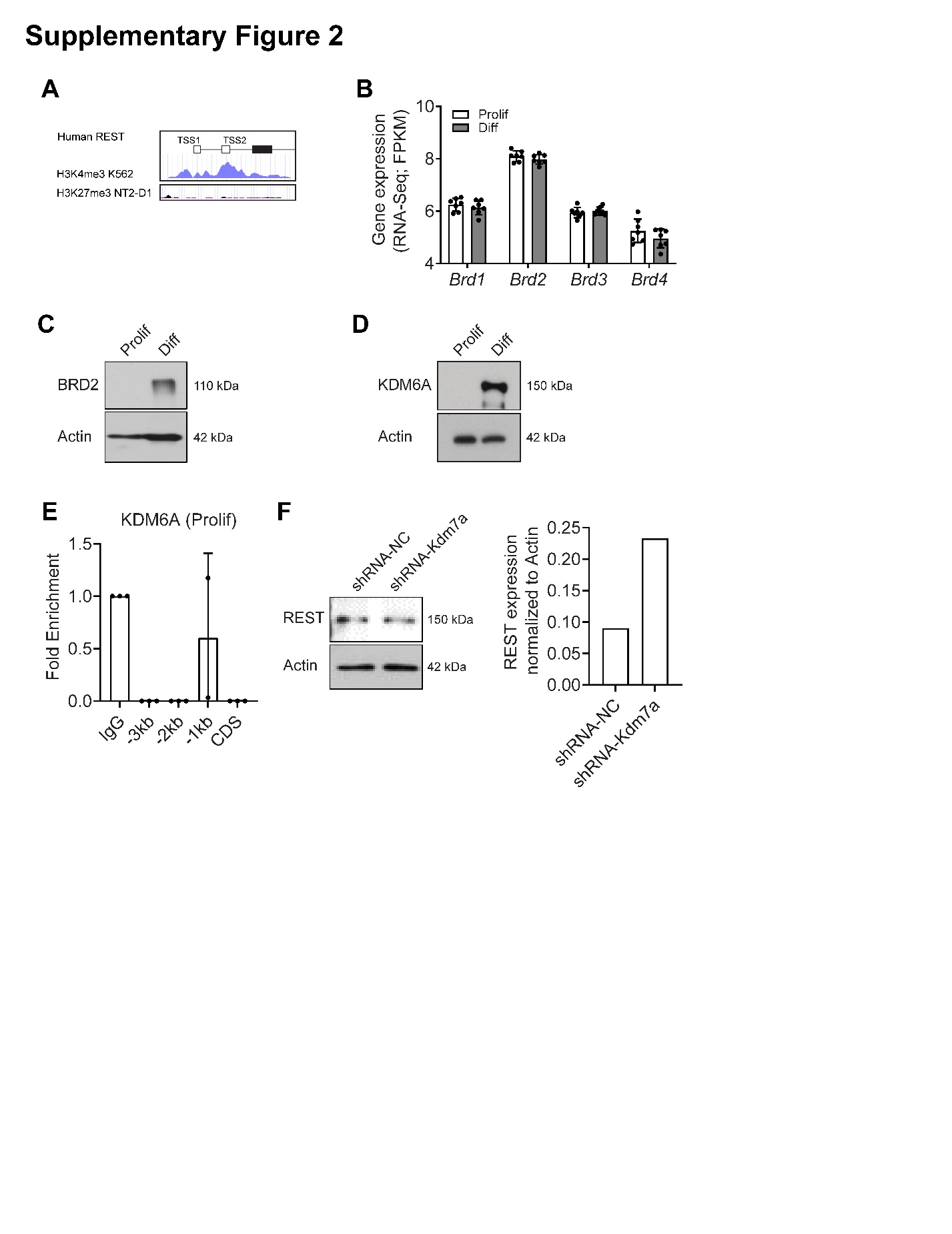
**

**
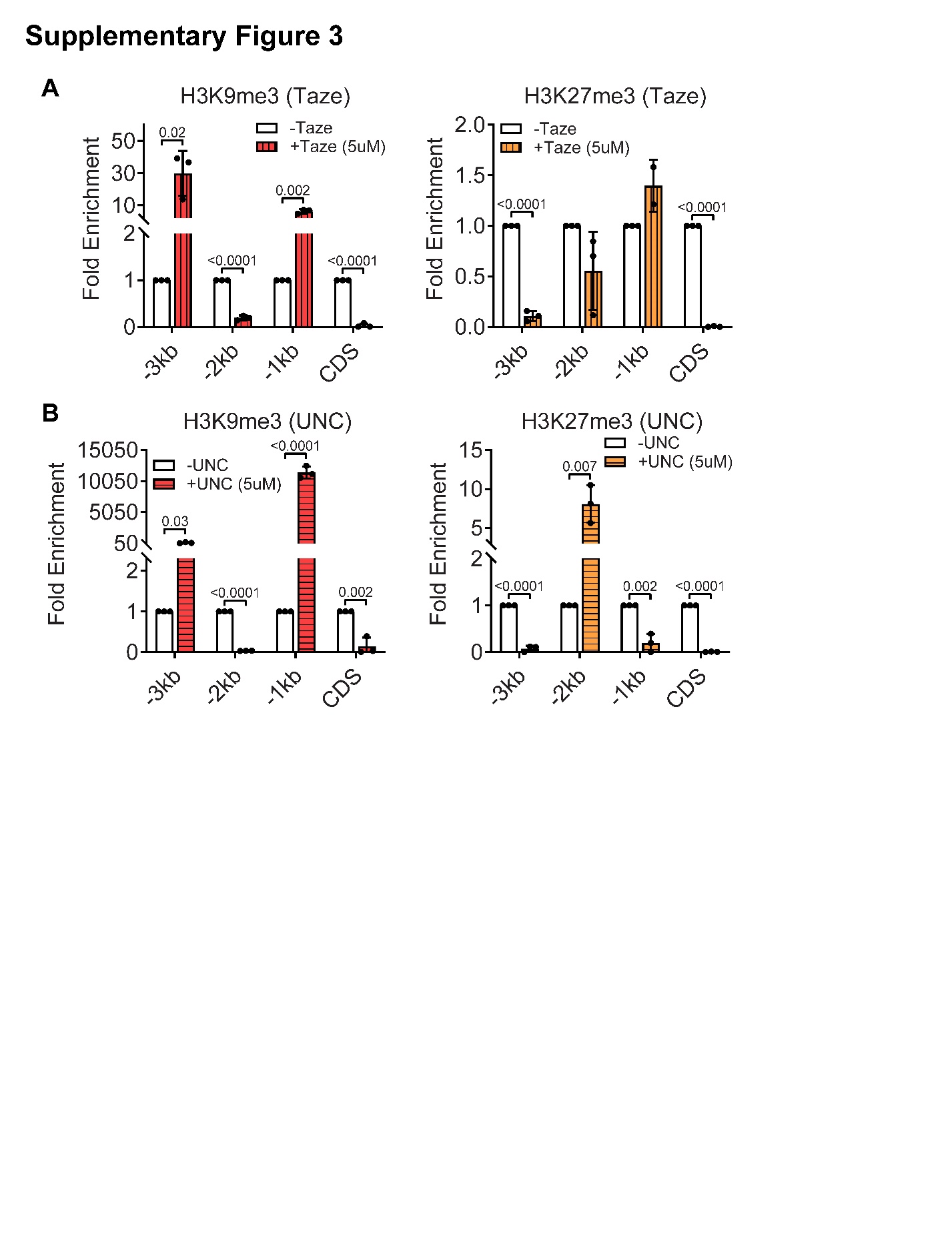
**

**
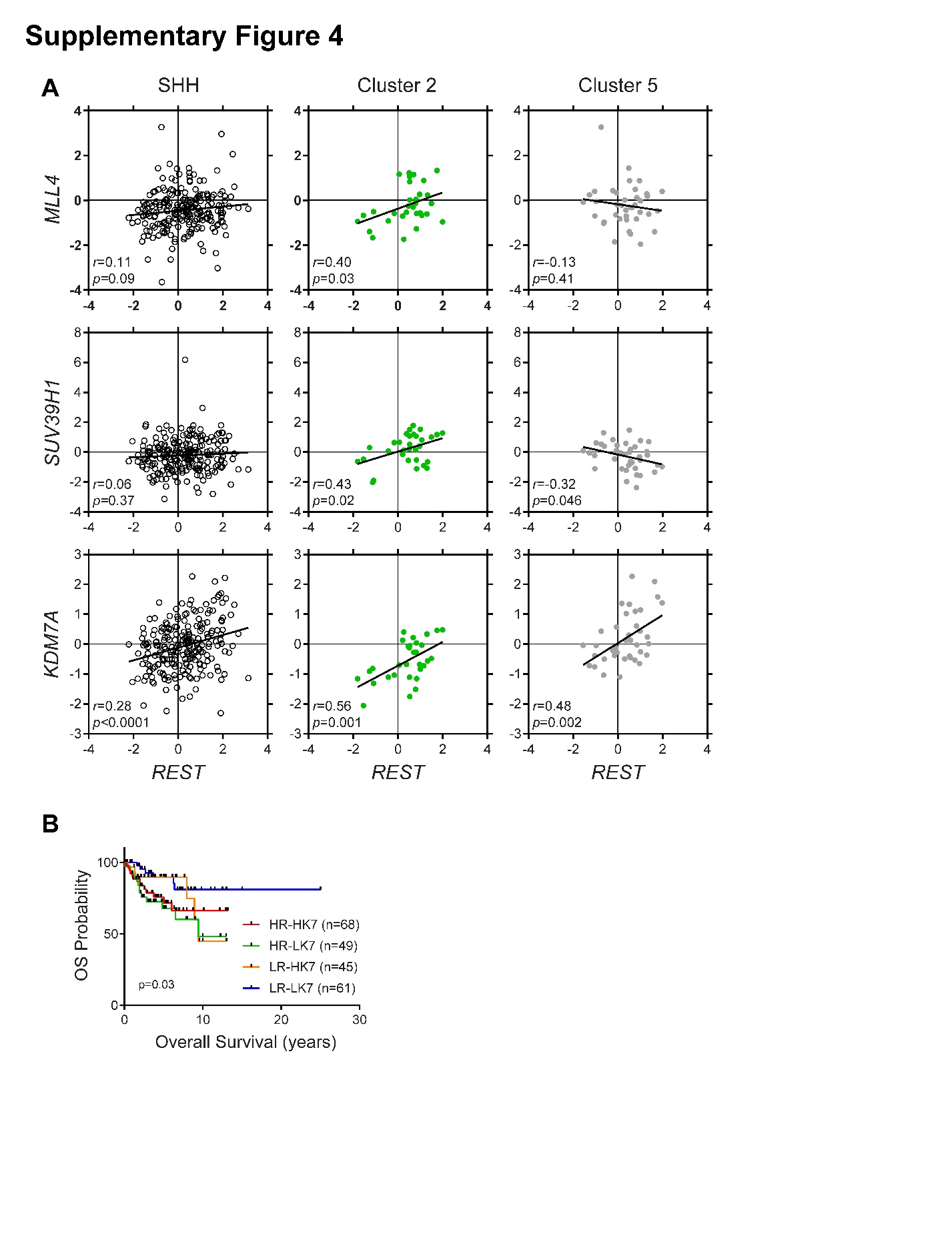
**

**Supplementary Figure 1 |**

**(A)** Cerebellar granular neural progenitors (CGNPs) harvested from P8 mice were cultured either in proliferating or differentiating media giving rise to neurospheres and differentiated neurons respectively. Representative images (n=3) are shown here. **(B, C and D)** Additional cerebellar sections from C57/Bl6 pups at P3, P8 and P12 were harvested, fixed and sectioned. Abundance of REST, KI67, and MAP2 was measured by IHCs using specific antibodies. Scale bars, 20μm. EGL; external granule layer, IGL; internal granule layer, PL; Purkinje layer, ML; molecular layer.

**Supplementary Figure 2 |**

**(A)** Schematic of *REST* locus on ENCODE using the UCSC browser in the K562 cells show high H3K4me3 at TSS and gene body and a basal level of H3K27me3 at the upstream promoter region in NT2-D1 cells. **(B)** Expression of *Brd1,2,3* and *4* measured by RNA-Seq (FPKM) of proliferating (white bars) and differentiating (gray bars) CGNPs. **(C)** Western blot analysis of BRD2 protein in proliferating and differentiating progenitors and Actin as loading control. Blot shown is a representative from n=2. **(D)** Western blot analysis of KDM6A protein in proliferating and differentiating progenitors and Actin as loading control. Blot shown is a representative from n=2. **(E)** Binding of KDM6A at the REST promoter was measured by ChIP-qPCR and shown as fold change over IgG in proliferating progenitors. **(F)** Kdm7a or a control shRNA was used for knock-down and Western blot of REST and Actin was performed and is shown in proliferating progenitors followed by a densitometry analysis.

**Supplementary Figure 3 |**

**(A)** Changes in H3K9me3 and H3K27me3 were measured by ChIP-qPCR in DMSO and 5 μM Tazemetostat treated differentiating progenitors. Graphs were plotted by normalizing DMSO control as 1 (n = 3). **(B)** Changes in H3K9me3 and H3K27me3 were measured by ChIP-qPCR in DMSO and 5 μM UNC-0638 treated differentiating progenitors. Graphs were plotted by normalizing DMSO control as 1 (n = 3).

**Supplementary Figure 4 |**

**(A)** Correlation of *REST* with *MLL4*, *SUV39H1* and *KDM7A* in SHH patient samples and in Cluster 2 and Cluster 5 separately are shown. **(B)** Overall survival of four patient groups based on *REST* and *KDM7A* in patients with SHH MB (P value; log-rank Mantel-Cox test).
